# Multi-target drugs for the treatment of cognitive impairment and fatigue in post-COVID syndrome: focus on *Ginkgo biloba and Rhodiola rosea*

**DOI:** 10.1007/s00702-024-02749-3

**Published:** 2024-02-12

**Authors:** Juliane K. Mueller, Walter E. Müller

**Affiliations:** 1 Department of Psychiatry, Psychosomatic Medicine, and Psychotherapy, University Hospital Frankfurt, Frankfurt/M, Germany; 2 Department of Pharmacology and Clinical Pharmacy, Goethe University Frankfurt, Frankfurt/M, Germany

**Keywords:** Post-COVID, Fatigue, Cognitive impairment, *Ginkgo biloba*, *Rhodiola rosea*

## Abstract

Cognitive impairment, depression and (mental) fatigue represent the most frequent neuropsychiatric symptoms of the post-COVID syndrome. Neuroinflammation, oxidative stress and mitochondrial dysfunction have been identified as common pathophysiological mechanisms underlying these symptoms. Attempts to treat post-COVID-associated cognitive impairment and fatigue with different drugs available for other diseases have not yet been successful. One probable explanation could be that these drugs work by one specific mechanism of action only and not in a broad multi-target way. Therefore, they will not address the broad pathophysiological spectrum possibly responsible for cognitive impairment, depression and fatigue in post-COVID syndrome. Notably, nearly all drugs currently under investigation for fatigue in post-COVID syndrome are rather addressing one single target instead of the several pathomechanisms underlying this condition. Contrary to this approach, herbal drugs often consist of many different ingredients with different pharmacological properties and pharmacological targets. Therefore, these drugs might be a promising approach for the treatment of the broad symptomatic presentation and the pathophysiological mechanisms of cognitive impairment and fatigue following a SARS-CoV-2 infection. Of these herbal drugs, extracts of *Ginkgo biloba* and *Rhodiola rosea* probably are the best investigated candidates. Their broad pharmacological spectrum in vitro and in vivo includes anti-oxidative, anti-inflammatory, antidepressant as well as properties reducing cognitive impairment and fatigue. In several studies, both drugs showed positive effects on physical and mental fatigue and impaired cognition. Moreover, depressive symptoms were also reduced in some studies. However, even if these results are promising, the data are still preliminary and require additional proof by further studies.

## Neuropsychiatric symptoms as important long-term sequelae of COVID-19

Due to an infection with SARS-CoV-2 *(severe acute respiratory syndrome coronavirus 2)*, COVID-19 (coronavirus disease 2019) can affect the whole body causing pulmonary, cardiovascular, hematological, gastrointestinal, nephrological, musculoskeletal, and neurological disorders (Bowe et al. 2022; Davis et al. 2023). Similar to other virus infections, COVID-19 is additionally associated with a large spectrum of neuropsychiatric symptoms during the acute clinical manifestation of the infection or shortly after (Rogers et al. 2020, 2021; Taquet et al. 2021a; Han et al. 2021). These neuropsychiatric symptoms include anxiety and depressive disorders but also less specific symptoms like sleep disturbances, cognitive deficits, and (mental) fatigue. While most of these symptoms resolve after weeks or a few months, they can persist in certain cases. Along with somatic symptoms, like pulmonary complications and physical fatigue, these symptoms may lead to long-term impairment of the affected patients. As the post-COVID syndrome (PCS) is defined as symptoms persisting longer than 12 weeks after the SARS-CoV-2 infection, persisting symptoms occur in up to 10% of SARS-CoV-2-positive patients, mainly in adults (Quan et al. 2023). Interestingly, the risk of developing PCS does not depend on the severity of COVID-19, as even patients with a symptom-free course of a SARS-CoV-2 infection can get affected by PCS. Similar to the acute COVID-19 phase, the clinical presentations of PCS might range from a variety of somatic symptoms to neurological and psychiatric symptoms (Taquet et al. 2021b; Sudre et al. 2021; Subramanian et al. 2022; Davis et al. 2023). Psychiatric symptoms associated with PCS are often the first episode of a psychiatric disorder and are most troublesome, especially for young patients (Badenoch et al. 2021; Tang et al. 2022; Kubota et al. 2022; Koczulla et al. 2022). Further, the neuropsychiatric symptoms of PCS often have a major impact on the distress of the patients. While many psychiatric sequelae (depression, anxiety, sleep disturbances etc.) can already be present during the initial phase of COVID-19, they can persist further on as part of a PCS often accompanied by fatigue or symptoms of fatigue. The post-COVID fatigue is causing a decrease in physical and/or mental performance. Typically, the patients report of exhaustion and fatigue, even after a sufficient period of rest. Importantly, the mentioned exhaustion and worsening of symptoms is not a result of massive physical or mental activity but rather following every day activities, not having caused any problems before the SARS-CoV-2 infection. This further impacts post-COVID patients in their daily activities and most important quality of life.

However, there is still controversy over the diagnostic specificity and duration of fatigue (Corfield et al. 2016; Joli et al. 2022). And even though many patients with PCS experience substantial suffering and impairment as mentioned above (Wong and Weitzer 2021; Kedor et al. 2022), they do not fulfill the diagnosis criteria of chronic fatigue syndrome (CFS) or myalgic encephalomyelitis/chronic fatigue syndrome (ME/CSF) (CDC 2021).

## Fatigue and cognitive impairment in post-COVID-syndrome: evidence for a common pathophysiology

Besides affective symptoms and sleeping disorders, cognitive impairment and fatigue are not only common symptoms in PCS (Quan et al. 2023; Chen et al. 2022; Joli et al. 2022; Calabria et al. 2022; Ceban et al. 2022a; Crivelli et al. 2022), but also seem to overlap as shown in some studies (Ceban et al. 2022a; Hartung et al. 2022). Similar findings about a high percentage of patients suffering from fatigue and deficits of memory, attention, and executive functions following different virus infections were already reported many years ago in the study by Joyce et al. (1996). The study by Azcue et al. (2022) showed similar results in patients with post-COVID-associated chronic fatigue syndrome or patients with Myalgic Encephalomyelitis/Chronic Fatigue Syndrome (ME/CFS) as both patient groups presented cognitive deficits (“brain fog”). A similar overlap of symptoms of fatigue and cognitive impairment in PCS and in ME/CFS was also reported in the review by Wong and Weitzer (2021).

Even if the pathophysiology of fatigue is not finally understood, substantial evidence suggests that oxidative stress, mitochondrial dysfunction, and subsequent chronic inflammation play a major role (Booth et al. 2012; Filler et al. 2014; Morris et al. 2019; Han et al. 2021). These findings have recently been confirmed by a proteomic analysis using peripheral blood mononuclear cells of ME/CFS patients (Sweetman et al. 2020) and post-COVID patients with ME/CFS (Paul et al. 2021). Thus, chronic inflammation including elevated levels of inflammatory cytokines and oxidative stress might also be relevant for post-COVID associated fatigue (Kedor et al. 2022; Al-Hakeim et al. 2021; Butler et al. 2022; Azcue et al. 2022; Mueller et al. 2022; Hadrawi et al. 2022; Lyra E Silva et al. 2022; Stefano et al. 2022). Furthermore, it seems that the brain is specifically sensitive to chronic inflammation (Baumeister et al 2022; Kappelmann et al. 2021). In addition to the above-mentioned overlap between cognitive impairment and fatigue, there is also a substantial overlap of depressive symptoms or even depressive disorders with fatigue in PCS. Again, chronic inflammation might also play a major role as an underlying common pathomechanism (Dabrowska et al. 2021; Al-Hakeim et al. 2021; Butler et al. 2022; Almulla et al. 2022; Lyra E Silva et al. 2022).

A similar role of inflammation for the symptomatic overlap of fatigue and depression has been reported for multiple sclerosis (Brys et al. 2020; Heitmann et al. 2022).

There seems to be an overlap of fatigue, depression, and cognitive impairment in PCS, suggesting a partially common but more importantly complex and multifactorial pathophysiology, with manifest symptoms often getting clinically apparent only after weeks or even months following the initial infection.

Further, the idea of a complex pathophysiology might explain why many available, but “repurposed” drugs (usually working via a rather specific molecular mechanisms of action) have not been successful for the treatment of post-COVID-associated fatigue. None of these available drugs address the multifactorial pathophysiology of post-COVID including chronic stress, oxidative stress and neuroinflammation causing fatigue, cognitive impairment (“brain fog”) and even symptoms of depression. Moreover, most if not all, drugs which are currently being evaluated in ongoing clinical trials as a possible post-COVID treatment in general or in specific for fatigue and cognitive impairment in PCS (Ceban et al. 2022b; Chee et al. 2023) address only one of the many targets probably relevant for the complex pathophysiology of PCS.

Further evidence for the multifactorial pathophysiological concept of PCS involving oxidative stress, mitochondrial dysfunction, and reduced energy supply comes from a recent study about beneficial effects of the nutritional supplement oxaloacetate in post-COVID (Cash and Kaufman 2022), although the study is currently under investigation due to questions about the methodology. Oxaloacetate is an energy metabolite which is reduced in the plasma of ME/CSF patients (Germain et al. 2017). Supplementation with oxaloacetate seems to reduce inflammation, increases the number of mitochondria, increases glucose uptake, and shows antioxidant activity in experimental studies (Wilkins et al. 2014; Li et al. 2022; Cash and Kaufman 2022).

## Are herbal multitarget drugs a therapeutic option?

Usually, synthetic drugs are developed to address one specific symptom or even one specific target only and do not fulfill the criteria of a multitarget drug or even more of a multifunction drug. However, this concept can be different for many medical herbs which usually comprise different compounds with different pharmacological profiles. Therefore, they may address different targets and further may have beneficial effects on different aspects of complex diseases (Luo et al. 2019; Zaa et al. 2023). However, in most cases, these medical herbs have been used as different, poorly defined extracts with very different compositions and concentrations of the active ingredients making any clear interpretation for the use in patient impossible. This was discussed in two reviews of the possible use of ginseng to improve cognition or fatigue, in which the many different ginseng preparations and the different dosages made a general conclusion about effectiveness impossible (Geng et al. 2010; Zhou et al. 2022).

Accordingly, we concentrated on the few herbal drugs available for possible treatment of post-COVID made with standardized extracts and with controlled preclinical and clinical evidence for therapeutic effectiveness. Two herbal drugs whose properties fulfill these criteria for the possible treatment of post-COVID-associated fatigue, cognitive impairment, and even symptoms of depression, are the standardized extract of *Ginkgo biloba* leaves (EGb761®) (Singh et al. 2019; Müller et al. 2019) and two standardized extracts of the roots and rhizome of *Rhodiola rosea* (SHR5, WS® 1375); which represent the most relevant and best investigated species of the so-called adaptogens (Panossian et al. 2021; Ivanova Stoijcheva and Quintela 2022; Anghelescu et al. 2018).

### Ginkgo biloba

*Ginkgo biloba* represents one of the best investigated herbal drugs available. In most experimental and clinical studies published over the last decades, a standardized extract (EGb761®^)^ was used with 6% terpenoids (ginkgolides and bilobalide) and 24% flavonoid glycosides (quercetin, kaempferol, isorhamnetin, etc.) as major active ingredients (Singh et al. 2022). The extract shows anti-oxidative and anti-inflammatory properties, improves mitochondrial function, and has been shown to increase many aspects of impaired neuroplasticity (Akanchise et al. 2023, Baliutyte et al. 2014; Singh et al. 2019; Müller et al. 2019; Müller et al. 2012).

Additionally, EGb761® has shown to improve cognitive function in a large number of animal studies. Further, EGb761® showed positive effects on cognitive impairment in humans over the whole spectrum of age-associated memory disorders from normal aging over mild cognitive impairment to neurodegenerative and vascular dementia (Müller et al. 2019). EGb761® is specifically active in reducing neuro-inflammation and behavioral sequelae of lipopolysaccharide (LPS)-induced neuro-inflammation in animal experiments (Zhao et al. 2015; Yeh et al. 2015). Interestingly, LPS treatment probably represents one of the most relevant animal models to induce symptoms of chronic fatigue as a consequence of prolonged activation of the immune system by illness or disease (Foster et al. 2021; Roth et al. 2021). In line with these results, EGb761® was effective in reducing depressive-like behavior in LPS-induced neuro-inflammation in rats (Yeh et al. 2015; Zhao et al. 2015) and showed positive effects on cognitive impairment due to oxidative stress and neuro-inflammation (Müller et al. 2019; Achete de Souza et al. 2020). EGb761® also has been shown to decrease the levels of serum cytokines in men including Il-6 (Mousavi et al. 2022), a major player in COVID-19 (Müller et al. 2022, Wang et al. 2022). Moreover, EGb761® improved impaired cognition by chronic inflammation (Wan et al. 2016; Liu et al. 2015).

Still, so far only few studies have presented evidence for a beneficial effect of *Ginkgo biloba* extract on fatigue. There are older results examining the effect of *Ginkgo biloba* extract on fatigue associated with multiple sclerosis (MS). Fatigue and cognitive impairment are known complications of MS, with a major impact on patients’ quality of live (Manjaly et al. 2019). Similar to the PCS, neuro-inflammatory mechanisms seem to play a major role (Manjaly et al. 2019).

In this study by Johnson et al (2006), 22 multiple sclerosis patients were treated with placebo or EGb761® 240 mg per day. Moderate but significant improvement over placebo was see on the MFIS (modified fatigue impact scale) and on two scales specifically developed for MS patients, the Functional Assessment of Multiple Sclerosis (FAMS), and the Symptom Inventory (SI). Although this is only a small study, the reported positive effects of EGb761® are in line with the multi-target pharmacology of this herbal drug.

In line with these results are findings by Zifko et al. (2022), about a case series of 5 patients with typical post-COVID symptoms like fatigue and cognitive deficits still present several months after the SARS-CoV-2 index infection. Patients were treated with EGb761® 160 mg/day for up to four months. All patients showed improvement in the area of fatigue and/or of cognitive impairment as measured by the clinical presentation as well as the Clinical Global Impression Severity scale (CGIC) and the Montreal Cognitive Assessment (MoCA). EGb761® was well tolerated. The author concluded that this drug may be a low-risk option to treat these post-COVID symptoms when given over many weeks.

Thus, the variety of pharmacological properties of EGb761® may fit the treatment requirements for fatigue/post-COVID-syndrome caused by a complex pathophysiology.

### Rhodiola rosea

Besides several other *Rhodiola* species, *Rhodiola rosea* is the best investigated member of the so-called adaptogens. Adaptogens are used in several countries to increase the resistance to a large number of pathological situations as stress, exhaustion, cancer, as well as viral and bacterial infections (Panossian et al. 2020; Anghelescu et al. 2018). *Rhodiola rosea* extract and its main constituent salidroside have been shown to improve cognitive deficits in a large number of experimental settings, and to show anti-ischemic effects, anti-inflammatory properties following liposaccharide induced inflammation, antioxidative properties, and improved neuroplasticity (Liu et al. 2015; Guan et al. 2012; Agapounda et al. 2022; Kumar et al. 2019). Further, its positive effects on learning and memory function as well as its antidepressant effects are well documented (Ma et al. 2018; Kumar et al. 2019; Zhong et al. 2019). Notably, the pharmacological properties of *Rhodiola rosea* show a substantial overlap with *Ginkgo biloba*.

So far, most of the experimental and clinical studies published for *Rhodiola rosea* over the last twenty years were carried out with two standardized dry extracts from the roots and the rhizome of *Rhodiola rosea* with somewhat different properties. SHR-5 (extraction with ethanol 70%, drug/extraction ratio 4.0:1.0, specified contents: 3.0–8.0% rosavins and > 1% salidroside) and WS® 1375 (extraction with ethanol 60%, drug/extraction ratio 1.5–5.0:1, specified contents 4.0% rosavins and > 2.5% salidroside) (Melzig et al. 2019). The broad pharmacology of *Rhodiola rosea* and its broad clinical profile as well as its major active ingredient salidroside have recently been reviewed by Ivanova Stojcheva and Quintela (2022) and Zhong et al. (2019).

Asthenia, resilience as well as physical and mental performance (the classical adaptogenic targets) were improved in several older studies using the extract SHR-5 as reviewed by Hung et al. (2011). Similarly, Edwards et al. (2012) showed in an open study with 100 patients that many stress-related symptoms improved after a 4-week treatment with WS® 1375.

WS® 1375, a standardized *Rhodiola rosea* extract showed beneficial effects on different cognitive measurements in a study with 50 adult participants after 6 and 12 weeks of treatment (Koop et al. 2020).

Also, positive effects on general fatigue were reported by Darbinyan et al., (2000) in a group of young, healthy physicians on night duty when treated with SHR-5 over several weeks. In an earlier study by Darbinyan et al. (2007), *Rhodiola rosea* L. extract showed anti-depressive efficacy. Similarly, patients with burnout-related symptoms showed significant improvement when treated with WS® 1375 for 8 or 11 weeks (Goyvaerts et al. 2012; and Kasper and Dienel 2017).

Furthermore, *Rhodiola rosea* showed positive effects on symptoms of physical and/or mental fatigue in several older studies as summarized by Ishaque et al. (2012), although the outcome of these studies was mixed. Five studies reported positive effects on impaired physical performance following acute stress with very little if any positive effects on endurance in healthy volunteers. Darbinyan et al. (2000) investigated the effect of *Rhodiola rosea* on mental fatigue in physicians on night duty. Again, the outcome was mixed with small positive effects on some cognitive measure. Probably the best of these older studies (Olsson et al. 2009) investigated 60 patients treated with 400 mg daily of *Rhodiola rosea* extract SHR-5. Compliance was rather poor. Primary outcome was evaluated with the Pines Burnout scale which showed a larger improvement in the verum group compared to placebo. Moreover, significant improvement over placebo was seen for several cognitive parameters on the CPT II scale (omissions, HIT RT, HIT RT SE, Variability). Salivary cortisol following the first hour after awakening was reduced in the verum group.

These findings seem to be in accordance with an open-label exploratory study by Kasper and Dienel (2017). In this study, 118 outpatients with burnout symptoms were treated with the standardized rhodiola extract WS® 1375. A majority of symptoms improved over the treatment time of 12 weeks including fatigue.

In a more recent study, 100 patients with chronic fatigue symptoms were treated with *Rhodiola rosea* for 8 weeks (Lekomtseva et al. 2017). Treatment effects were evaluated using several standard scales including the multidimensional Fatigue Inventory, the Clinical Global Impression scale, and the numbers connection test. In this open-label study, substantial improvement was seen on all measures suggesting positive effects on physical as well as mental fatigue. Since the improvement seen in this study was much more pronounced than placebo responses in general reported for many other studies on fatigue (Cho et al. 2005), the authors suggest that the substantial effects observed in this study on nearly all measures cannot be explained by placebo effect only. Still these results have to be noticed with caution.

No therapeutic effect was seen in the study by Punja et al. (2014), in which 50 nurses on night duty reporting of fatigue were treated with *Rhodiola rosea* for several weeks. However, even the authors suggest to see the findings with caution due to several problems with the design of the study, the patient randomization, changes from protocol, small sample size, and the use of a self-made *Rhodiola rosea* extract of unknown specifications and quality.

So far, studies about a possible effect of *Rhodiola rosea* extract on post-COVID-associated fatigue and other neuropsychiatric symptoms have not yet been published, although the possible use of *Rhodiola* in post-COVID fatigue has been suggested (Wegener et al. 2023).

There is only one double-blind placebo-controlled study investigating the effect of ADAPT-232, a herbal tonic containing *Rhodiola rosea* extract, Eleutherococcus and Schisandra extracts, in post-COVID patients (Karosanisze et al. 2022). ADAPT-232 is available as a food supplement in several countries. And even though, the compounds have adaptogenic properties (Panossian and Wikman 2010), regarding ADAPT-232 the final composition, stability, and properties are not well understood. In the study by Karosanisze et al. (2022), 100 patients with post-COVID symptoms within the last 30 days before entering the study were treated with placebo or ADAPT-232 for two weeks only. Post-COVID patients were defined as having a minimum of 3 out of the following nine symptoms (fatigue, headache, respiratory insufficiency, cognitive performance, mood disorders, loss of smell, taste and hair, sweatiness, cough, and pain in joints, muscle and chest). While all patients of both groups improved over time (observation period only two weeks), only daily walk time and measures of respiratory insufficiency showed significant improvement over placebo. At the endpoint, there was a significant decrease of serum creatinine and a small decrease of cytokine Il-6 in the verum group.

These rather disappointing results are in contrast to the positive effects of *Rhodiola rosea* on fatigue in previous studies as reviewed by (Ivanova Stojcheva and Quintela 2022). Possibly this is due to major differences in respect to the rather high doses of dried *Rhodiola* extracts used in the other studies. The dosage of *Rhodiola rosea* extract in the ADAPT-232 preparation is rather low and has already been shown to be ineffective in reducing experienced fatigue and stress in a previous study. Interestingly, a *Rhodiola* extract alone in a much higher dosage (used as a positive control) was effective in this study by Schutgens et al. 2009. Moreover, many other factors may have contributed to the rather negative findings in the study by Karosanidze et al. (2022). Several drawbacks of design and performance of the study raise doubts about the validity of the data like the use of a mixture of three liquid extracts which are not really stabilized, the rather short treatment time, the rather diffuse inclusion criteria (only three of nine post-COVID symptoms were required). Moreover, treatment was only for two weeks while much longer treatment periods had been used in most other (positive) studies.

### Lavandula angustifolia

A specific preparation of lavender oil (Silexan®) is available in many countries as liquid gelatine capsules as an anxiolytic drug (Kasper et al. 2018). Preclinical and clinical evidence indicates efficacy to treat anxiety disorders and mixed anxiety/depression (Müller et al. 2021; Bartova et al. 2023). As mixed anxiety/depression is seen in post-COVID patients, the possible therapeutic usefulness of Silexan® in these patients has been discussed and seems to be supported by a series of case reports (Dold et al. 2023; Kasper et al. 2023).

## Final conclusions

Cognitive impairment, depression and fatigue are common, partly overlapping symptoms of the post-COVID-syndrome. Oxidative stress, mitochondrial dysfunction, and neuro-inflammation are common pathophysiological factors underlying these symptoms. Many available drugs for other disorders with similar symptoms have failed so far to show relevant therapeutic effects for these post-COVID symptoms probably because of the multifactorial pathophysiology of the post-COVID syndrome. Contrary to most synthetic drugs, usually working by one specific mechanism of action, many herbal drugs consist of different compounds with different pharmacological properties. Several of these herbal drugs show antioxidant, anti-inflammatory and cognition improving properties. The most prominent herbal drugs are *Ginkgo biloba* and *Rhodiola rosea*. Extracts of both herbal drugs fulfill the preclinical multifactorial requirements to improve symptoms of cognitive impairment and fatigue in post-COVID syndrome by showing antioxidant, cognition improving, neurorestorative, anti-inflammatory, and antidepressant properties. The preclinical findings are supported by many clinical studies about positive effects on symptoms of mental and physical fatigue in affected patients. The quality of these studies however is mixed. Only few studies have already been published about beneficial effects on fatigue and cognitive impairment in post-COVID patients. In summary, both herbal drugs address many of the discussed pathophysiological mechanisms possibly underlying the PCS. Therefore, both may have a positive effect on symptoms of fatigue and cognitive impairment in the course of the PCS, especially when given over several weeks. However, even if these results are promising, especially considering the benign side effect profile of both substances, the data available so far are still preliminary and require additional confirmation  by further studies in post-COVID syndrome.
